# Age Differences in Motor Recruitment Patterns of the Shoulder in Dynamic and Isometric Contractions. A Cross-Sectional Study

**DOI:** 10.3390/jcm10030525

**Published:** 2021-02-02

**Authors:** Cristina Lirio-Romero, Rocío Palomo-Carrión, Helena Romay-Barrero, Asunción Ferri-Morales, Virginia Prieto-Gómez, María Torres-Lacomba

**Affiliations:** 1 Faculty of Physiotherapy and Nursing, Physiotherapy and Occupational Therapy Department, University of Castilla-La Mancha, Avda, Carlos III s/n, 45004 Toledo, Spain; Cristina.Lirio@uclm.es (C.L.-R.); Asuncion.Ferri@uclm.es (A.F.-M.); 2 GIFTO Research Group, Avda, Carlos III s/n, 45004 Toledo, Spain; 3 Physiotherapy in Women’s Health (FPSM) Research Group, Physiotherapy Department, Faculty of Medicine and Health Sciences, University of Alcalá, Alcalá de Henares, 28805 Madrid, Spain; v.prieto@uah.es (V.P.-G.); maria.torres@uah.es (M.T.-L.)

**Keywords:** shoulder, musculoskeletal disorders, surface electromyography, age groups, aging

## Abstract

Aging processes in the musculoskeletal system lead to functional impairments that restrict participation. Purpose: To assess differences in the force and motor recruitment patterns of shoulder muscles between age groups to understand functional disorders. A cross-sectional study comparing 30 adults (20–64) and 30 older adults (>65). Surface electromyography (sEMG) of the middle deltoid, upper and lower trapezius, infraspinatus, and serratus anterior muscles was recorded. Maximum isometric voluntary contraction (MIVC) was determined at 45° glenohumeral abduction. For the sEMG signal registration, concentric and eccentric contraction with and without 1 kg and isometric contraction were requested. Participants abducted the arm from 0° up to an abduction angle of 135° for concentric and eccentric contraction, and from 0° to 45°, and remained there at 80% of the MIVC level while isometrically pushing against a handheld dynamometer. Differences in sEMG amplitudes (root mean square, RMS) of all contractions, but also onset latencies during concentric contraction of each muscle between age groups, were analyzed. Statistical differences in strength (Adults > Older adults; 0.05) existed between groups. No significant differences in RMS values of dynamic contractions were detected, except for the serratus anterior, but there were for isometric contractions of all muscles analyzed (Adults > Older adults; 0.05). The recruitment order varied between age groups, showing a general tendency towards delayed onset times in older adults, except for the upper trapezius muscle. Age differences in muscle recruitment patterns were found, which underscores the importance of developing musculoskeletal data to prevent and guide geriatric shoulder pathologies.

## 1. Introduction

Aging leads to a regression of physical capacities and a decrease in functionality in older people [1,2]. Around 35% of people over 65 years of age suffer from neuromuscular disorders [3,4]. The proportion of older people will increase in the coming decades [5], so establishing normative data in order to improve diagnosis, prevention, and treatment is a necessity to reduce costs due to absence of information.

In mid-adulthood, age-related changes appear that progressively decrease muscle function [1,6]. These natural aging changes are clinically defined as geriatric sarcopenia [7,8]. With age, the muscle mass in skeletal muscle decreases, especially for type II fibers, which lead to a decrease in strength, with a consequent increase in muscle weakness [9]. In addition, neuronal factors are also altered and are responsible for the loss of muscle function [10].

Regarding upper limbs, shoulder muscle function decreases with less use of arms and hands in daily activities. Furthermore, it appears to impair balance, which increases the risk of falls mainly in older adults [11]. The typical recruitment pattern of shoulder muscles has already been studied in healthy adults [12], as well as their differences between the sexes [13]. Furthermore, as previously mentioned, the literature shows physiological changes with age [7,9,14]. However, no evidence has been found linking these changes with possible differences in motor recruitment patterns. Therefore, it is essential to understand age-related physiological alterations from different activities of the upper limb, such as dynamic and isometric movements involved in daily tasks—previous subjects of study in the lower limb [15,16].

Abduction is the shoulder movement commonly used for function evaluation because it gives useful information on the control and quality of the movement of the upper limb [17]. The scapula is in a favorable position during abduction since metabolic energy has not been required by passive scapulothoracic forces [18]. Analysis of muscle recruitment patterns has been used through surface electromyography (sEMG) to understand functional differences in muscles recruitment [19,20]. sEMG is also used by physical therapists to better understand the function and dysfunction of the neuromusculoskeletal system [20].

The present study aims to describe muscle recruitment patterns during dynamic and isometric contractions of abduction movement to identify age-related muscle function. The objective was to provide electromyography data on motor recruitment during shoulder abduction in adults and older adults, both healthy, to facilitate a preventive or therapeutic approach to loss of upper limb function.

Additionally, the implication of possible motor disorders of the shoulder complex muscles in older people on the loss of force, mobility, and functionality of the upper limb is intended to be observed.

## 2. Materials and Methods

### 2.1. Design

A descriptive cross-sectional study in two age groups was carried out (Registry: NCT04706169) [21], in which the sEMG activity (amplitude and onset) of the middle deltoid (MD), upper trapezius (UT), infraspinatus (IS), lower trapezius (LT), and serratus anterior (SA) was compared in adults and older adults.

All study participants were informed about the purpose of the study, signed the informed consent, and participated voluntarily. The Ethics Committee “Clinical Research of the University of Alcalá (Madrid, Spain)” approved the study (2012/038/01/20,120,924).

### 2.2. Participants

Participants attended, from December 2015 to March 2019, the laboratory of the Research Group “Physiotherapy in Women’s Health Research Group”, at Teaching and Research Unit in Physiotherapy of the University of Alcalá (Madrid, Spain) and Ocaña Senior Center (Toledo, Spain) voluntarily after reading an advertisement about the need to recruit healthy people for a research study. A physical therapist (C.L.-R.), experienced and trained in sEMG recordings, performed the assessment.

Participants, without symptoms in the shoulder and/or cervical area during the last year, were assigned to the respective age groups: over 65 years (Older adults) and 20 to 64 years (Adults). Participants with rheumatological diseases, moderate or severe cognitive impairment, tumors, massive osteoarthritis, circulatory disorders, dermatological problems, sedentary people, or those who had received physiotherapy within the 12 months prior to sEMG assessment were excluded from the study as well as those who took medication that could have repercussions in motion processing.

### 2.3. Assessments/Interventions

sEMG was used to measure the amplitude and onset of five shoulder muscle activities performing glenohumeral abduction. In this movement, the middle deltoid muscle was selected because it is a main motor. The infraspinatus muscle represented the rotator cuff muscle group. The middle deltoid muscle was selected as the representative of shoulder abduction because it is a main motor in this movement [22]. The trapezius muscle and especially the serratus anterior muscle were chosen as representative, established of the ascending scapular rotator muscles [18].

For determination of the force values by means of the maximum isometric voluntary contraction (MIVC), necessary to normalize the signal and maintain the isometric contraction, the participants held a dynamometer (MicroFET^®^2, Hoggan Health Industries, West Jordan, UT, USA) [23]. To detect MIVC, the participants raised their arm to 45° of glenohumeral abduction. The handheld dynamometer was placed on the forearm at a medium distance between the wrist and the elbow. This position was marked to ensure the reliability of the dynamometer measurement during submaximal force tests. Next, the participant isometrically abducted his arm with maximum effort while the dynamometer was firmly fixed by the physical therapist (C.L.-R.). The participants repeated this three times, and the average value was used to determine the MIVC value.

The submaximal level of isometric contraction was determined at 80% of the MIVC. Prior to electromyographic evaluation, submaximal tests were performed. The physical therapist instructed the participants to perform an isometric glenohumeral abduction at 45° for 5 s using the hand dynamometer to mark the respective submaximal force level. To record the electrical activity during the submaximal isometric contraction, elevation of the arm was requested for 2 s in the abduction movement from 0° to 45° [24], and maintained for 5 s once they reached the value of 80% of their MIVC. The abduction displacement was recorded/registered by the electronic goniometer (MLTS700, ADInstruments, Oxford, UK).

In addition, a physiotherapist (C.L.-R.) trained with participants how to perform abduction movements up to 135° for 7 s and return to the starting position of 0° (without weight and with a 1 kg weight in hand). Subjects chose weight they thought they would lift in their normal daily activities (range = 1–3 kg) [25,26,27]. As for the older adults, they lifted the weight that allowed them to complete the range of movement (1 kg).

Once proofs had been performed and MIVC values were taken, electrodes were placed to record the activity of the five muscles, as well as the electric goniometer to record movement. The surface electromyograph used was a PowerLab 15T (ADInstruments, Oxford, UK). The experienced physical therapist placed sEMG electrodes for precise positioning. Conductive adhesive hydrogel surface electrodes (27 mm diameter) (Kendall^TM^ 100 series Foam Electrodes, Covidien, MA, USA) were used, using a 30 mm electrode gap. The skin was wiped with alcohol and two electrodes were placed on the midline of the respective muscle bellies, aligned along the muscle fibers. In addition, ground electrodes were placed on bone sites (processus spinosus C6, C7, and the posterior part of the acromion). The electric goniometer was positioned so that one sensor was fixed on the upper part of the scapula and the other on the back of the arm at a 90° angle between both sensors and preset in the 0° position (i.e., neutral position) in the sEMG registration software. Adhesive tape was used to fix the electrodes and cables. sEMG (gain: 1000) and goniometric signals were sampled at a rate of 1000 samples per second, using a 16-bit AD converter.

**Figure 1:**
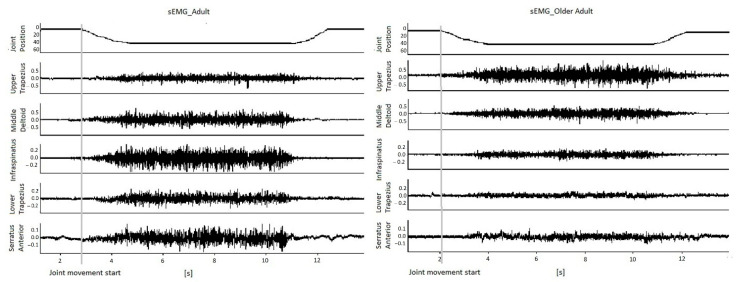
Raw surface electromyography (sEMG) recordings of the five muscles (indicated on the **left**) simultaneously displaying the abduction movement (from 0° to 45°). These have been obtained from one representative participant from the Older Adults group and one from the Adults groups, displaying the abduction movement.

The sEMG signals were band-pass filtered (10–500 Hz, eighth Bessel filter) to improve the signal-to-noise ratio. LabChart^®^ Software (ADInstruments, Oxford, UK) was used to simultaneously capture sEMG data on a PC. Root mean square (RMS) values were obtained automatically, within the time interval of 2 to 4 s after the start of the contraction and were normalized according to the respective MIVC. Muscle onset values were obtained from analysis graphs that included the arm displacement recorded simultaneously with sEMG (Figure 1). The onset was obtained as the time distance of the interception between the level of pre-activation relative to the onset of arm displacement during dynamic contraction and the linearly interpolated RMS slope [12,19].

To obtain reliable electromyographic signal data, we have tried to reduce crosstalk, motion artifacts, skin contact impedance, and power supply noise by correct electrode placement and filters [19].

sEMG data of all the investigated muscles were simultaneously recorded (Figure 1). They were requested to perform (a) 3 repetitions of glenohumeral abduction up to 135° and return to position 0, (b) 3 equal repetitions, but with a 1 kg weight in their hand, and (c) 3 repetitions of isometric abduction (intervals between tests of 2 min). The mean sEMG values of the 3 repetitions were used for further analysis.

Demographic variables were collected: age, sex, dominant body side, height and weight (body mass index), and physiotherapy treatments within the 12-months prior to sEMG assessment. This last issue was registered to understand any possible variation despite the healthy condition of all subjects during the last year. The MIVC, glenohumeral range of motion (ROM), and the Shoulder Disability Questionnaire were analyzed as clinical variables. As was previously reported, we determined the MIVC at 45° of abduction [28] by a hand dynamometer. These data served as reference values to normalize the sEMG signal and to determine the levels of submaximal isometric contraction previously reported.

Glenohumeral ROM was measured with a universal goniometer (Enraf Nonius Ibérica^®^, Madrid. Spain): flexion, internal and external rotation, and abduction. To assess possible shoulder dysfunction, although with no complaints, the Shoulder Disability Questionnaire was used. The Shoulder Disability Questionnaire is widely used in research and clinical practice in several countries. It consists of 16 items about shoulder complaints during tasks performed in the last 24 h (yes, no, or not applicable). The ratio of the number of items with an affirmative answer over the number of applicable items was multiplied by 100. Scores range from 0 (no functional limitation) to 100 (affirmative to all items); higher scores mean higher disability [29].

A sEMG variable result was the amplitude of muscle activity quantified through the normalized RMS. Additionally, the onset(s) of muscle contraction was registered/recorded during dynamic contraction. Therefore, the time between the start of the abduction movement and the start of the contraction of each muscle was calculated [19,30].

### 2.4. Sample Size

We determined the sample size considering differences in the levels of MIVC between the two groups of 30 participants each. Assuming within the group a standard deviation of 20.9 N, a standard deviation of 30.1 N could be detected in the ANOVA between groups with type I error of 0.05 and 80% power. Furthermore, to detect group differences, a difference of 21 N was reported assuming identical power and variations within the group [13].

### 2.5. Statistical Analysis

Data were analyzed using IBM SPSS Statistics 20 for Windows (SPSS Inc., 2011, IBM Corp, Armonk, NY, USA). The Shapiro–Wilks test was used to test normal or non-normal distribution of variables. The mean and standard deviations, as central tendency measures, were estimated in the normal distributed variables, and the median and interquartile range in the not normally distributed variables. Student’s *t*-test and Mann–Whitney U test were used to calculated significant differences between adults and older adults. A 95% confidence interval for each estimator was used.

## 3. Results

**Figure 2:**
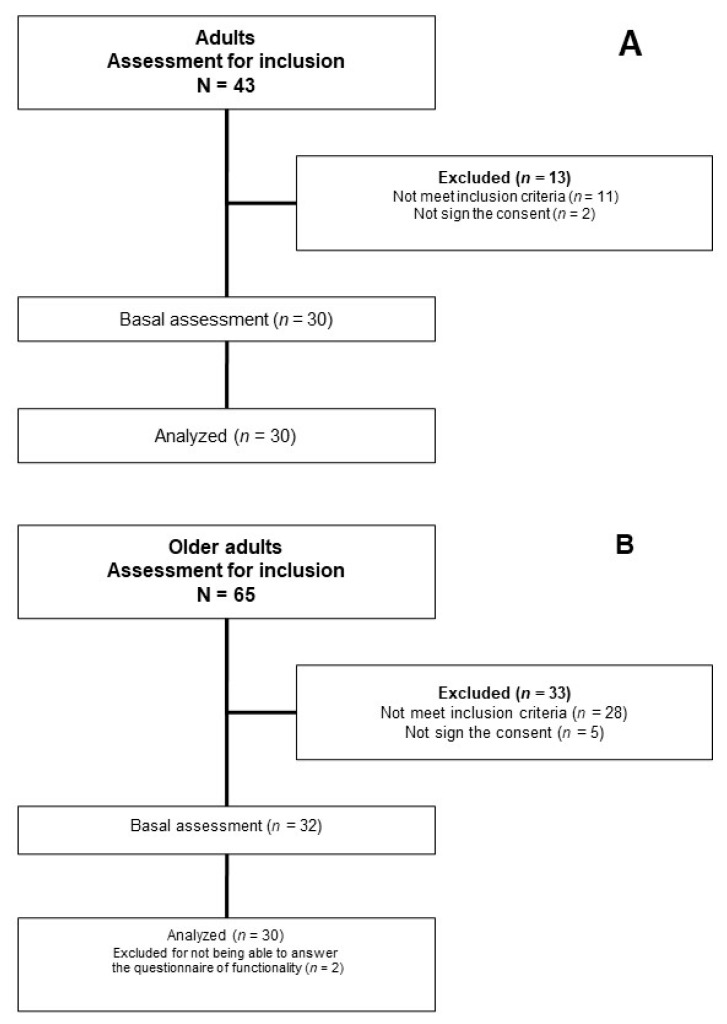
Flow of participants. (**A**) Adults; (**B**) Older adults.

The flow chart shows the process for selecting participants (Figure 2).

### 3.1. Age-Related Differences in Demographic and Clinical Data

Statistically significant differences were observed between age groups in functionality and body mass index (*p* < 0.01, Adults < Older adults) as well as in MIVC (*p* < 0.01, Adults > Older adults), active ROM *p* < 0.05, Adults > Older adults), and previous physiotherapy treatments (*p* < 0.05, Adults < Older adults). Respective values are shown in Table 1 for each group.

### 3.2. Age-Related Differences in sEMG Signal

**Figure 3:**
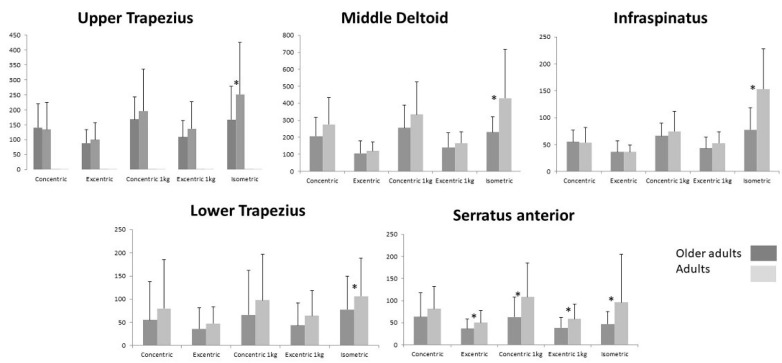
Means (standard deviation) of RMS for each muscle are shown for every muscle contraction: dynamics (concentric and eccentric; with and without 1 kg resistance) and isometric. * *p* < 0.05 between age groups.

In general, the RMS values showed a decrease in older subjects (Older adults) with respect to adults in all muscles analyzed in terms of the three types of contraction (concentric, eccentric, and isometric) with and without added weight. However, no statistically significant differences were found in dynamic contractions regardless of load (Figure 3). Statistically significant differences were only found for isometric contraction (*p* < 0.05). In addition, the SA muscle showed statistically significant differences in terms of eccentric contractions with or without weight and concentric with weight (*p* < 0.05, Adults > Older adults).

The rest of the analysis referring to concentric and eccentric contractions with or without weight did not show statistically significant differences (*p* > 0.05), although a decrease in the amplitude of the sEMG signal of all contractions was observed in the Older adults group (Figure 3).

**Figure 4:**
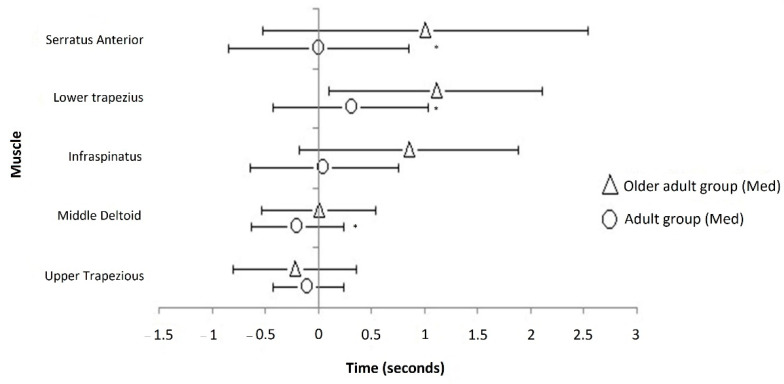
Onset time of muscles contraction. The onset of concentric contraction was averaged for each muscle during shoulder joint abduction for all subjects in Adults and Older adults. Median (±RI) values for each muscle are shown. The vertical line at zero seconds represents the start of the abduction movement. The anticipated contraction is showed to the left (negative) of the zero line. * *p* < 0.05 between age groups.

Regarding onset times, the tests performed showed in all muscles analyzed during glenohumeral abduction that they progressively delayed with age with the exception of the UT muscle, which showed an advance for Older adult group (Figure 4). The delay observed between age groups was significantly different for the scapula stabilizers, LT and SA (*p* < 0.01, adults < Older adults) and for DM and IS (*p* < 0.05, Adults < Older adults). In general, a different order of recruitment between age groups was observed. Not observed in adults, the UT muscle was the first one to be recruited in older adults. UT muscle was recruited before the beginning of the abduction movement compared with the adults group that showed later UT contraction (*p* > 0.05)

## 4. Discussion

The present study compared the motor recruitment patterns of shoulder muscles between the adults and older adults groups during dynamic and isometric glenohumeral abduction, observing differences in isometric (against high resistance) and especially SA muscle in both types of contractions. The sEMG amplitude and onset time of contractions were the primary outcome measures. Older people showed lower sEMG amplitudes during abduction compared to the adults group. With increasing age, the onset of glenohumeral abduction contraction in most of the times analyzed was found to be delayed in older people. Differences between age groups were found in shoulder ROM, body mass index, shoulder function, and previous physiotherapy treatments. The decrease in shoulder functionality has previously been related to age [31], and to loss of muscle mass and range of motion [17].

To understand the age-related changes found in MIVC levels, it is not possible to contrast these data with sEMG amplitude [32], but it is necessary to consider degenerative alterations in the composition, size, and number of muscle fibers [33], and the replacement of the contractile structure by connective tissue or fat [34]. The loss of fibers is related to the decrease in the number of motor neurons with age [14]. Plow et al. tried to explain the loss of motor neurons and the alteration in the number of fibers because of changes in the motor cortex with age but did not obtain such findings [35].

### 4.1. sEMG Signal

As with previous studies [12,13,36], differences in sEMG amplitude with age are indicative of differences in the patterns of recruitment and its consequent tendency towards functional alteration. In opposition to the results found in the isometric contraction, the dynamic contractions (concentric and eccentric) did not show statistically significant differences between age groups. This variation in EMG signal between types of contraction has previously been reported [15,37].

Starting from the planning of the contractions, it is necessary to clarify that it was intended to imitate the muscular action that the muscles analyzed perform in daily activities. Everyday tasks require dynamic actions that use more easily achievable static forces [38]. Dynamic contractions are commonly performed over wide ranges of motion and with no or light load (especially in older people), and isometric contractions are frequently performed against resistance. In addition, posture, together with impaired coordination of the scapular musculature, are factors that influence the strength of dynamic contractions and reduce the range of motion [39]. Knowing the functioning of contractions is key to understanding the greater significance in the results of submaximal isometric contraction, where higher motor units are recruited. In fact, during isometric contractions, motor units generally show higher recruitment thresholds than in dynamic contractions [40]. However, we can deduce that when evaluating a healthy population with symptoms, we can see that age is not a relevant factor in dynamic contractions, but rather in requests for isometric contractions against submaximal resistance. However, the disadvantages previously reported in the normalization of the sEMG signal in different age groups [41] prevent us from affirming that the submaximal isometric contraction changes more with age than the dynamic contractions.

Focusing on submaximal isometric glenohumeral abduction at 45°, the UT, LT, and SA muscles function as stabilizers of the scapula [18]. It is worth highlighting the results regarding the differences between age groups found in SA that show a tendency to lose scapular stability with age, which in turn explains the loss in the last degrees of glenohumeral abduction, although without clinical symptoms, and loss of shoulder function in the healthy older population. Shoulder pathologies also have reported similar motor patterns of decreasing SA muscle activity [42,43]. Since the activity of the SA depends not only on force production but also on neuromuscular control and recruitment, a precise coordinated activity may occur at the right moment. This proper firing pattern and recruitment requires coupling of the serratus anterior muscle with the trapezius that results in “force couples”, necessary for normal scapular orientation [44]. Non-appropriate activity of these muscles in older adults could depend on lower proprioception, more effort and heaviness associated with the muscular activity, and worse perceived timing of muscle contraction with aging [45]. SA muscle recruitment deficits in older adults have been manifested by its altered pattern of recruitment or its altered timing (delayed muscle onset), which can reduce shoulder movement. Given that, only significant differences were evidenced in dynamic contractions in the SA muscle. It can be assumed to be the most altered muscle found in older adults under healthy conditions, which is of interest to understand the functional deterioration of the upper limb with age. However, as a low correlation between the SA muscle signals has been found using surface and intramuscular electrodes, sEMG to assess muscle activation levels in the SA muscle is not the best option [46], also considering the possible influence that higher body mass index may exert, thereby disturbing data recordings in older adults [47].

The onset was significantly different between adults and older adults in all muscles analyzed as shown by previous studies [48], except for UT muscle. The delay in the start of contraction with the normal aging process has been explained by neuromuscular impairments in transmission or by muscle weakness. Kwon et al. relate this delay with an alteration in the co-activation of the antagonist musculature with age [49]. As suggested by Kibler [50,51], the co-activation of the upper trapezius and serratus anterior hold the activation of the rotator cuff muscles. The balance between the glenohumeral internal and external rotators may be compromised by the alteration in upper trapezius and serratus anterior recruitment patterns, which also could be related to less shoulder function in older adults. However, it is not possible to understand why there was more significant differences in all glenohumeral ROM, except for internal rotation. Differences with age in movement are not the result of a decrease in force or speed, but rather central factors that affect movement coordination [52,53].

The present study was able to demonstrate that the main stabilizing muscle of the scapula showed the most notable decrease in the amount of recruitment in addition to a delay tendency in the onset of contraction in older people. Its known tendency towards reduction in scapular stability is pointed out as the basis of certain shoulder disorders. As has been shown in previous evidence [13,43], UT muscle anticipation could compensate the delay of SA and LT muscles. Moreover, possible latent trigger points in the muscle analyzed could be present in subjects of both groups. In an asymptomatic population, latent trigger points could alter the muscle activity signal. Regarding muscle contraction onset, a large variability in the onset times displayed has previously been observed for muscles with latent trigger points [27].

The present arguments hope to provide a tool for prevention and treatment of geriatric disorders, paying special attention to the prevention of loss of activity of the scapular stabilizer muscles.

### 4.2. Limitations and Clinical Implications

The study design considered the inherent problems in measuring sEMG. The greater difficulty in normalizing the signal in older people should be noted, as well as the non-normalization of the sEMG signal muscle by muscle, but in general for the five muscles according to the MIVC. This was performed in this way to avoid loss of interest mainly of the participants of the older adults group.

Furthermore, the great variability in sEMG results between subjects limits their analysis. This may be due to individual factors in degree of physical activity and hormonal or pharmacological factors, which could have been recorded. We found a high variability when referring to the sEMG values in the reviewed studies and latent trigger points must be considered in following studies about muscle activity differences. This made the discussion and interpretation of the data presented more difficult.

Important age-dependent differences have been identified in the motor recruitment patterns of the shoulder muscles, especially for high resisted isometric contractions, showing that they undergo alterations with age, especially in relation to scapula stabilization and coordination between SA and UT muscle. Healthcare professionals can use the present findings for prevention or treatment; given the great difficulty that exists to carry out activities against high resistance as one common musculoskeletal complain in geriatric diseases. It is a wake-up call to attention to the tending deterioration of the SA muscle.

## 5. Conclusions

The results showed age differences in muscle recruitment patterns in static maintenance of shoulder lift, but not in its dynamic contraction, which can influence loss of force, range of movement, and functionality even in a healthy population, emphasizing the serratus anterior as the more challenging muscle and its tendency to reduce its activity. This underscores the importance of developing an early treatment approach from adulthood to prevent and guide geriatric shoulder pathologies. This is intended to awaken interest in launching new studies that evaluate possible therapeutic approaches that address the desynchronization found in older shoulder muscles.

## Figures and Tables

**Table 1:** Demographic and clinical characteristics.

Variables	Adults *n* = 30	Older Adults *n* = 30	*p*-Value ^†^
Age Median (IR *)	45.5(27.5)	70.5(8.3)	<0.01
Gender *n* women (%)	15(50)	16(53.3)	0.80
Dominant limb *n* right (%)	30(100)	28(93.3)	0.16
Previous physiotherapy treatments *n* yes (%)	2(6.7)	11(36.7)	<0.05
Body mass index Mean (SD **)	24.4(3)	29.2(3.6)	<0.01
MVIC [Newton] Mean (SD **)	122.9(44)	75.7(26)	<0.01
SDQ Median (IR *)	0(0)	0(12.5)	<0.01
Glenohumeral Flexion Mean (SD **)	160.5(9.5)	151.2(9.3)	<0.01
Glenohumeral Internal Rotation Mean (SD **)	73.8(10.6)	70.7(22.7)	0.49
Glenohumeral External Rotation Mean (SD **)	85.5(8.4)	71.8(15.1)	<0.01
Glenohumeral Abduction Mean (SD **)	160.8(11.8)	147.5(9.5)	<0.01

SDQ—Shoulder Disability Questionnaire; * IR—Interquartile Range; ** SD—Standard Deviation; ^†^ *p*-value obtained by Mann–Whitney U test, χ^2^ test and Student *t*-test.
